# Collision of ductal adenocarcinoma and neuroendocrine tumor of the pancreas: a case report and review of the literature

**DOI:** 10.1186/s12957-017-1157-9

**Published:** 2017-05-02

**Authors:** Simone Serafini, Gianfranco Da Dalt, Gioia Pozza, Stella Blandamura, Michele Valmasoni, Stefano Merigliano, Cosimo Sperti

**Affiliations:** 1 Department of Surgery, Oncology and Gastroenterology, 3rd Surgical Clinic, University of Padua, via Giustiniani 2, 35128 Padua, Italy; 2 Department of Pathology, University of Padua, Padua, Italy

**Keywords:** Collision tumor, GIST, Intraductal papillary mucinous tumor, Neuroendocrine tumor, Pancreatic cancer

## Abstract

**Background:**

Simultaneous occurrence of exocrine and neuroendocrine tumors of the pancreas is very infrequent. We report a patient with an endocrine tumor in the pancreatic-duodenal area and extensive exocrine carcinoma involving the whole pancreas.

**Case presentation:**

A 69-year-old woman was hospitalized in May 2016 for epigastric pain and weight loss. Her past medical history revealed an undefined main pancreatic duct dilation that was subsequently confirmed at CT scan (23 mm) and endoscopic ultrasound. There was no evidence of pancreatic masses, but the cephalic portion of the main pancreatic duct presented hypoechoic nodules. A diagnosis of the main-duct intraductal papillary mucinous neoplasm was made, and the patient underwent total pancreatectomy. Pathological examination showed a collision tumor constituted by a ductal adenocarcinoma involving the whole pancreas and a neuroendocrine tumor located in the duodenal peripancreatic wall and the head of the pancreas. There was one peripancreatic lymph node metastasis from the ductal adenocarcinoma and eight node metastases from the neuroendocrine tumor. These findings suggested a diagnosis of collision of neuroendocrine and ductal adenocarcinomas of the pancreas. The postoperative course was uneventful.

**Conclusions:**

The coexistence of pancreatic endocrine and exocrine tumors is very uncommon. When present, problems in differential diagnosis may arise between mixed exocrine-endocrine carcinoma or the collision of separate tumors.

## Background

Simultaneous occurrence of a pancreatic exocrine and endocrine tumor (neuroendocrine tumor (NET)) is very infrequent. In large series studies, the incidence of combined neoplasms ranged only from 0.06 to 0.2% of all pancreatic tumors [1, 2]. Collision cancers are defined as tumors located in the same organ or anatomic site. According to the World Health Organization (WHO) histological classification, collision tumors include at least two different malignant components, without mixed or transitional area [3]. In this study, we report a patient with a collision pancreatic tumor constituted by a pancreatic ductal adenocarcinoma (PDAC) and NET associated with a jejunal gastrointestinal stromal tumor (GIST). A review of the English literature was performed searching PubMed (MEDLINE) using “pancreatic neoplasms,” “pancreatic cancer,” “neuroendocrine tumor,” and “pancreatic collision tumor” as keywords. The related article function was used, and all abstracts, studies, and citations obtained were reviewed. To our knowledge, this is the first case of an extensive PDAC that collided with a NET.

## Case presentation

**Fig. 1:**
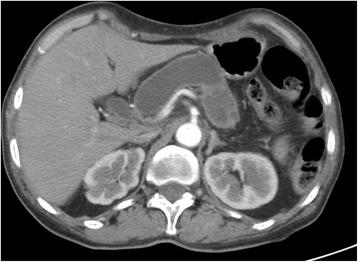
Abdominal CT scan showing the dilation of the pancreatic main duct

**Fig. 2:**
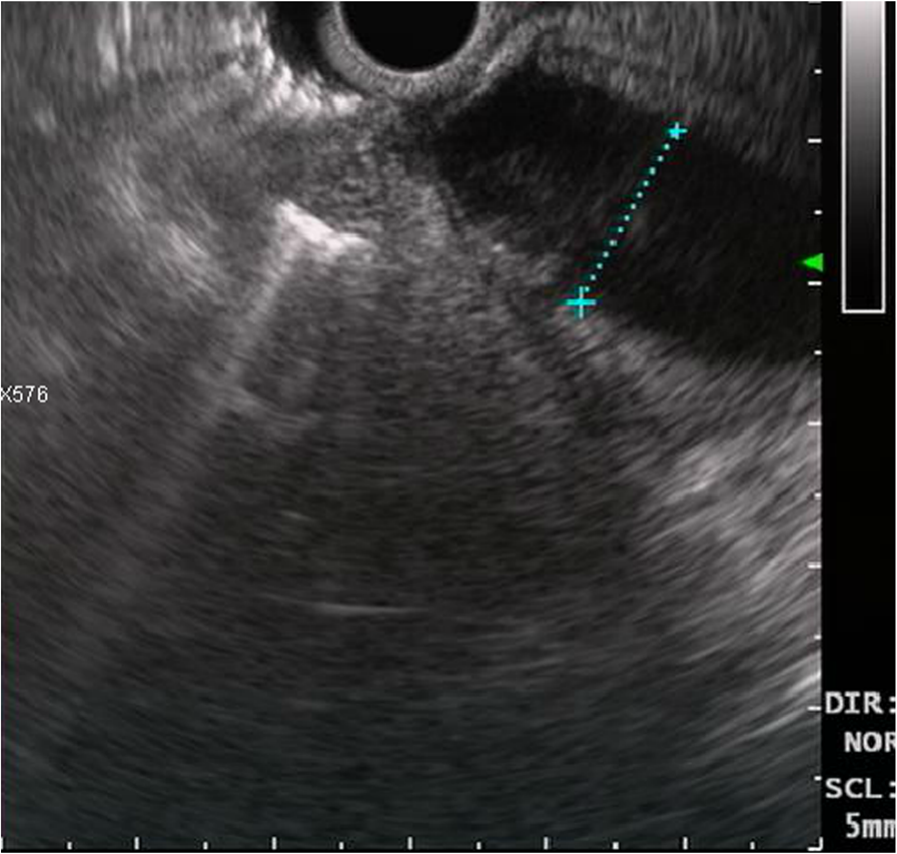
Endoscopic ultrasound showing a marked dilation of the pancreatic main duct

A 68-year-old Caucasian woman was admitted in May 2016 for the examination of a suspected intraductal papillary mucinous neoplasm (IPMN). Her previous medical history included breast cancer, diabetes mellitus, hypertension, hypercholesterolemia, and multiple congenital skeletal dysplasia. She presented with weight loss, epigastric pain, and fatigue. Laboratory examination revealed high carbohydrate antigen 19-9 (Ca 19-9) serum levels (139.9 U/mL; normal value <37 U/mL). Triple-phase computed tomography (CT) of the abdomen detected a dilated main duct (diameter of 23 mm from the head to the tail) without solid lesions (Fig. 1). Endoscopic ultrasound (EUS) confirmed main duct dilatation >20 mm, with mural hypoechoic nodules (Fig. 2). Malignant main-duct IPMN was suggested, and the patient underwent surgery. At laparotomy, neither liver metastasis nor peritoneal seeding were found. A solid 26-mm lesion in the jejunal wall was incidentally detected. So, total pancreatectomy and excision of the jejunal lesion were performed. The postoperative period was uneventful.

Macroscopic examination of the resected specimen showed a dilated main pancreatic duct (diameter of 20 mm for a length of 80 mm); an irregular, not well-defined mass (50 mm in longest diameter) in the body-tail of the pancreas; and a solid lesion in the jejunum.

**Fig. 3:**
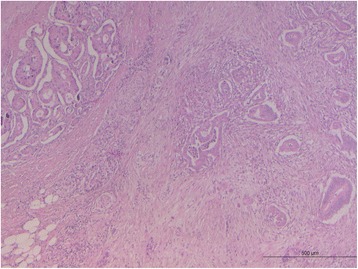
Pancreatic slide view of the collision tumor. The morphological differences between the two types of tumors are remarkable: NET on the *upper left side* and PDAC on the *right*

**Fig. 4:**
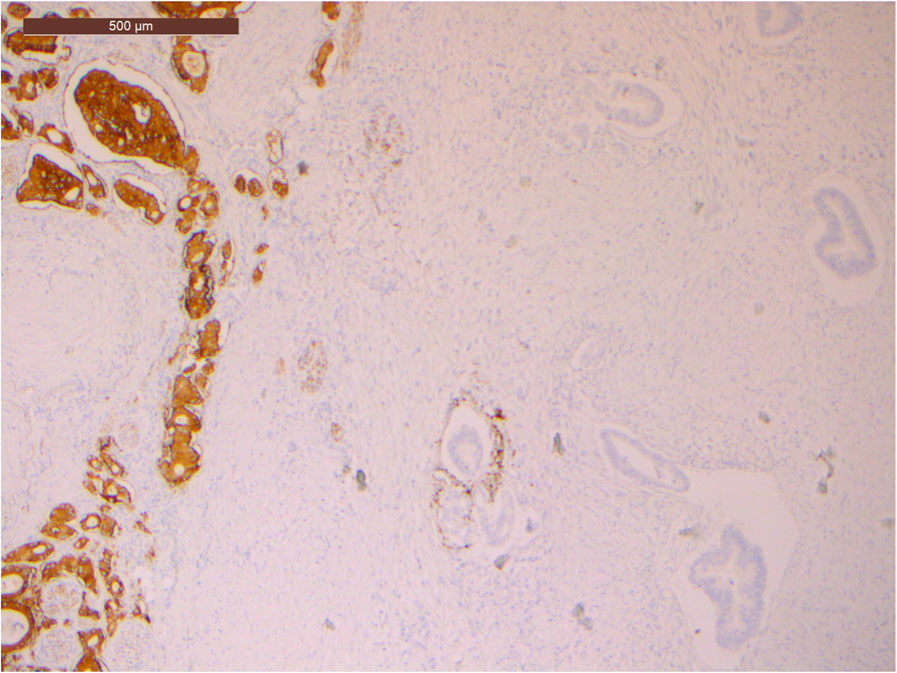
Immunohistochemical synaptophysin slide showing positive neuroendocrine cells

**Fig. 5:**
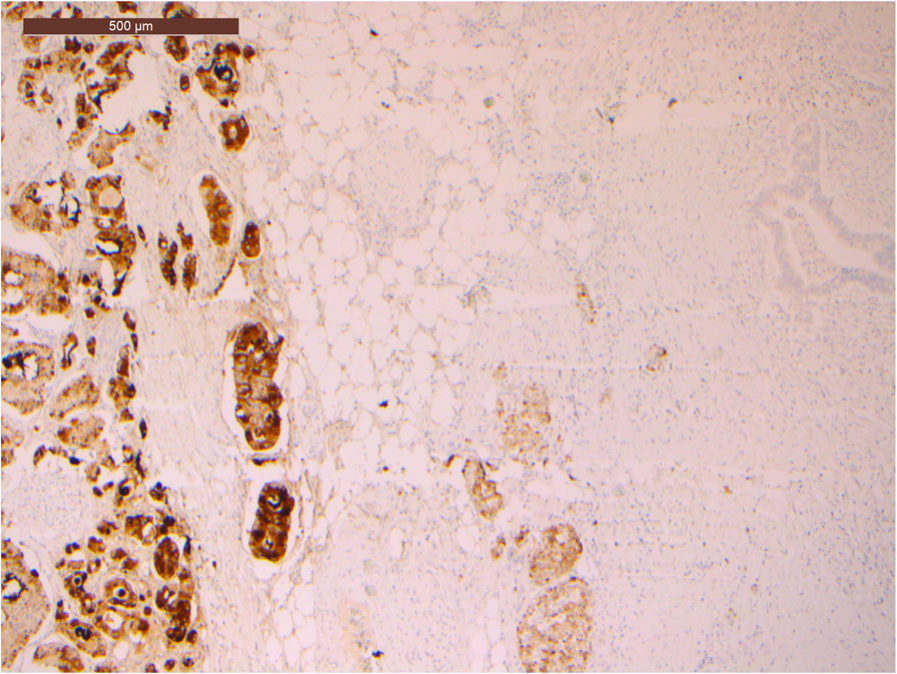
Immunohistochemical chromogranin A slide showing positive neuroendocrine cells

Microscopic sections showed a collision pancreatic tumor constituted by a ductal adenocarcinoma, including pancreatic intraepithelial neoplasia (PanIN), and a G1 NET, in the duodenal peripancreatic wall and the head of the pancreas (Fig. 3). The PDAC collided with the NET without mixing: the NET was localized in the periduodenal portion of the head of the pancreas reaching also the duodenal wall nearby Vater’s ampulla, while the exocrine component of the tumor involved the remaining pancreas, from the head to the tail. Lymphovascular invasion and perineural spreading were also detected. Thirty-nine regional lymph nodes were examined in the resected specimen with one node metastasis (peripancreatic node) from the PDAC and eight node metastases from the NET (seven peripancreatic nodes and one para-aortic node). The ductal differentiation was of conventional type, characterized by medium-sized glandular structures of variable shapes, embedded in desmoplastic stroma with foci of poor glandular differentiation found in the peripancreatic tissue. The neoplastic glands infiltrated the underlying duodenum. The endocrine component grew up in the head of the pancreas and in the duodenal wall reaching the submucosa, with signs of endo-lymphatic invasion. At immunohistochemistry analysis, endocrine cells were synaptophysin+ (Fig. 4), chromogranin A+ (Fig. 5), NSE +, and somatostatin+ (weakly) and showed a mitotic index of 1–2 mitoses × 10 HPF, a Ki-67 proliferation index of 2%, and frequent psammoma bodies. Reactions for gastrin, serotonin, insulin, glucagon, calcitonin, and pancreatic polypeptide were negative. Ductal dysplasia and carcinoma in situ, characterized by irregular epithelial budging and bridging, small papillae, lack of fibro-vascular stalks, and severe nuclear abnormalities, were detected in the pancreas and in the pancreatic main duct. Pathological examination of the jejunal lesion showed a fusiform type GIST, CD117+ and CD34+, and wildtype for c-KIT/PDGFRA.

After surgery, the patient refused gemcitabine-based adjuvant treatment. Currently, the patient has no signs of relapse 8 months after surgery.

## Discussion

We report the first case of a collision pancreatic tumor constituted by a PDAC, without features of IPMNs, and NET, associated with an incidental jejunal GIST. Preoperative work-up was consistent with malignant main-duct IPMN, due to the dilation of the pancreatic main duct (more than 2 cm), high serum Ca 19-9 levels, and patient’s symptoms. Surprisingly, pathological examination showed an extensive adenocarcinoma that collided with a neuroendocrine tumor involving the duodenum and the head of the pancreas, with metastasis at the peripancreatic and para-aortic nodes.

Collision tumors of the pancreas are rare; sporadic cases of concomitant or collision tumors constituted by IPMNs and NET [4–7], solid pseudopapillary neoplasm and NET [8], and cancer of the bile duct and pancreas [9] have been previously reported, but clinicopathological features and prognosis of these tumors are substantially unclear. The incidence of concomitant IPMNs and NETs has been reported in a range from 2.6 to 4.6%, suggesting a non-random association [10–12]. In most cases, the coexistence of both tumors was an incidental discovery after the examination of the surgical specimen. Two cases of a concomitant IPMN and a pancreatic NET diagnosed before surgery have also been reported [13]. In 2013, Ishida et al. [7] described a case of simultaneous IPMN and NET in the pancreas and collected 15 previously reported cases. Half of the patients were symptomatic (mainly abdominal pain), only one patient presented with hormone-secreting tumor, three patients had metastases from a NET, and one patient died due to a metastatic NET. The origin of the neoplastic population (common progenitor cell or random association) has not been explained. Many authors suggested that these tumors may arise from common precursor stem cells [10, 14–16] as showed by a recent molecular lineage study [17]. Others hypothesized that the carcinogenesis of collision cancer may lead to alteration of local immunodefence after the development of one tumor or to the effect of a carcinogenic agent able to affect different targets simultaneously [5]. Prognosis of collision malignant tumors is still unclear. In 2010, the largest experience of collision cancers based on ten heterogeneous cases of pancreatic and periampullary cancers was reported [5]. In this series, most collision cancers were IPMNs coexisting with other malignancies (PDAC, NET, lower end of common bile duct, and duodenal ampullary carcinoma) and showed poor prognosis with a median survival time of only 10 months.

Moriyoshi et al. [4] reported an interesting case of a collision pancreatic tumor constituted by extensive PanIN, coexisting with IPMNs with focal invasion and multiple NETs in a patient affected by multiple endocrine neoplasia type 1. No lymph nodes metastases were found; follow-up of the patient is not reported. Most of NETs concomitant with exocrine neoplasms are non-functioning tumors, but two cases of IPMNs and endocrine syndrome from nesidioblastosis (hypoglycemia) and vipoma (watery diarrhea), respectively, have been reported [18, 19].

In our case, the presence of psammoma bodies could suggest a somatostatin-producing NET; however, immunohistochemistry examination showed only a weak positivity for somatostatin and negativity for gastrin, serotonin, and pancreatic hormones. Moreover, the coexistence of a NET and a GIST suggests a possible type 1 neurofibromatosis, but the patient did not have clinical manifestations (such as cutaneous and ocular) or familial history of the disease.

Differential diagnosis between mixed exocrine-endocrine and collision tumors may arise. However, in the mixed type, the exocrine-endocrine cells are closely combined [20] while the collision type shows separate endocrine and exocrine components without an intermixed central zone [3], as in our case.

Survival of patients with an extensive adenocarcinoma and a malignant NET of the pancreas is obviously unknown. Our patient refused adjuvant therapy, but she is still alive and disease-free 8 months after surgery.

## Conclusions

In conclusion, we report a new case of adenocarcinoma coexisting with a metastatic NET of the pancreas, misinterpreted as a malignant IPMN. Intraoperative detection of a jejunal GIST also occurred. Based on our case and review of the literature, collision pancreatic cancer is a very uncommon tumor composed at least of two different malignant components. Pathogenesis of this rare entity is substantially unclear, and problems in differential diagnosis may arise between mixed exocrine-endocrine carcinoma or the collision of two distinct tumors. Preoperative diagnosis is difficult because of the lack of specific symptoms and radiological features. Radical resection is still the treatment of choice for resectable tumors, but the prognosis appears unpredictable.

## Abbreviations

GIST: Gastrointestinal stromal tumor
IPMN: Intraductal papillary mucinous neoplasm
NET: Neuroendocrine tumor
PDAC: Pancreatic ductal adenocarcinoma
WHO: World Health Organization.
